# A new species of open-air processional column termite, *Hospitalitermes nigriantennalis* sp. n. (Termitidae), from Borneo

**DOI:** 10.3897/zookeys.554.6306

**Published:** 2016-01-18

**Authors:** Syaukani Syaukani, Graham J. Thompson, Herbert Zettel, Teguh Pribadi

**Affiliations:** 1 Department of Biology, Faculty of Mathematics and Natural Science, Syiah Kuala University Darussalam 23111, Banda Aceh, Indonesia; 2 Department of Biology, University of Western Ontario, 1151 Richmond Road North, London N6A 5B7, Ontario, Canada; 3 Entomological Department, Natural History Museum Vienna, Burgring 7, 1010 Vienna, Austria; 4 Department of Forestry, Faculty of Agriculture, PGRI University of Palangkaraya, Central Kalimantan 73112, Indonesia

**Keywords:** Termite, Hospitalitermes, new species, central Kalimantan, Borneo

## Abstract

A new species of open-air processional column termite is here described based on the soldier and worker castes from eight colonies in north Barito, central Kalimantan. *Hospitalitermes nigriantennalis* **sp. n.** is readily distinguished in the field from related *Hospitalitermes* spp. by the light brown to orangish coloration of the soldier head capsule that, further, is with vertex yellowish and nasus brownish. The soldier antenna and the maxillary and labial palps are blackish. By contrast, soldiers from other species of *Hospitalitermes* from this region have a uniformly black head capsule and antennae. Finally, *Hospitalitermes nigriantennalis* **sp. n.** has a minute indentation in the middle of the posterior part of head capsule, which further helps to differentiate this new species from other *Hospitalitermes* from the Indo-Malayan and Austro-Malayan regions.

## Introduction

More than 2,900 living termite species (Engel et al. 2009) belonging to 281 genera have been described worldwide (Krishna et al. 2013). This diversity is partitioned among nine families, six of which (Kalotermitidae, Archotermopsidae, Hodotermitidae, Rhinotermitidae, Stylotermitidae, and Termitidae) are known from the Oriental region (Roonwal 1970). Three of these (Kalotermitidae, Rhinotermitidae and Termitidae) have been recorded in the Indo-Malayan sub-region of Asia (Ahmad 1965, Thapa 1981, Tho 1992).

The open-air processional column termites consist of three genera: *Hospitalitermes* Holmgren, 1912, *Lacessititernes* Holmgren, 1912 and *Longipeditermes* Holmgren, 1912 (Syaukani et al. 2011). Most species of this group are conspicuous because, unlike the vast majority of termites, workers and soldiers forage above ground or on leaf litter in processional columns (Tho 1992, Jones and Gathorne-Hardy 1995, Miura and Matsumoto 1998, Syaukani 2010, Syaukani et al. 2011). Almost all individuals are relatively quick moving, as evidenced by their disproportionately long legs. Further, they are heavily pigmented, which is presumably related to their above-ground lifestyle and camouflage on leaf litter (Jones and Gathorne-Hardy 1995). This three-genus group has been well described, and much is know about its distribution, caste system and feeding behavior (Kalshoven 1958, Roonwal 1970, Collins 1979, Jones and Gathorne-Hardy 1995, Miura and Matsumoto 1998, Jones and Brendell 1998).

Seven species have previously been recorded from Borneo: *Hospitalitermes hospitalis* (Haviland, 1898), *Hospitalitermes hospitaloides* (Holmgren, 1913), *Hospitalitermes rufus* (Haviland, 1898), *Hospitalitermes lividiceps* (Holmgren, 1913), *Hospitalitermes umbrinus* (Haviland, 1898), *Hospitalitermes flaviventris* (Wasmann, 1902) and *Hospitalitermes medioflavus* (Holmgren, 1913) (Krishna et al. 2013). In this paper the eighth species from this region is described.

## Material and methods

Specimens of *Hospitalitermes nigriantennalis* sp. n. were collected from a processional column on the primary forest floor in Pararawen Nature Reserve, Muara Teweh, North Barito, Central Kalimantan (Borneo), Indonesia. The head, body (in profile), pronotum and antenna of the soldier caste (preserved in 70% ethanol) were photographed using a digital microscope (Olympus SZX12 and Nikon DS-Fi2, Japan). From these images, multi-focused montages were constructed using Helicon Focus 6.2.2 software (Helicon Soft Ltd. Kharkov Ukraine). General morphological terminology used for describing soldiers and workers follow Roonwal and Chhotani (1989), Sands (1998), Tho (1992) and Syaukani et al. (2011).

Measurements of the soldier body parts specifically follow precedent from Roonwal and Chhotani (1989) and Tho (1992). Head capsule length including nasus (HLN), head capsule length excluding nasus (HL), nasus length (NL), nasus index = NL/HL, maximum head width at anterior part (HWA), maximum head width at posterior part (HWP), maximum height of head capsule excluding postmentum (HH), pronotum length (PL), and pronotum width (PW). Measurements for the soldier caste are summarized in Table 1.

**Table 1.** Measurements (in mm) for n = 20 soldiers of *Hospitalitermes nigriantennalis* sp. n. from eight colonies.

Character	Holotype	Size range
Head capsule length including nasus	1.72	1.64–1.72
Head capsule length excluding nasus	1.18	1.01–1.19
Nasus length	0.65	0.63–0.65
Nasus index	0.55	0.60–0.61
Maximum head width at anterior part	0.58	0.55–0.60
Maximum head width at posterior part	1.06	1.00–1.07
Maximum height of head capsule excluding postmentum	0.82	0.75–0.82
Pronotum length	0.37	0.32–0.37
Pronotum width	0.62	0.56–0.63

## Systematics

### Family Termitidae Latreille, 1802 Genus *Hospitalitermes* Holmgren, 1913

#### Hospitalitermes nigriantennalis

Taxon classificationAnimaliaIsopteraTermitidae

Syaukani & Thompson sp. n.

http://zoobank.org/1EEA9B9D-A871-41EB-A2F7-38055284D924

Figs 1–3 , 4–5 , 6–13 , 14 , 15

##### Description.

Imago. Unknown.

**Figures 1–3.**
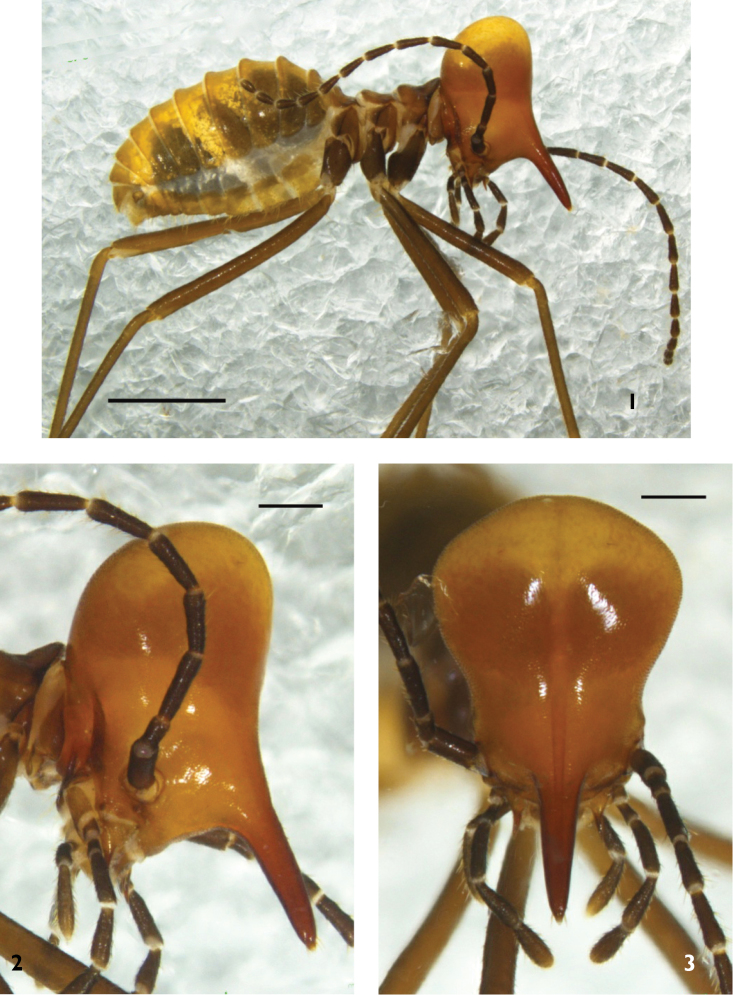
Soldiers of *Hospitalitermes nigriantennalis* sp. n. Habitus in profile (**1**), head in profile (**2**), head in dorsal view (**3**). Scale bars: 0.5 mm (**1**), 0.3 mm (**2, 3**).

**Figures 4–5.**
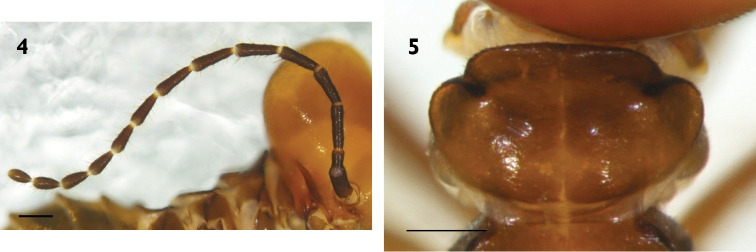
Soldier of *Hospitalitermes nigriantennalis* sp. n. Antenna (**4**) and pronotum (**5**). Scale bars: 0.3 mm (**4**), 0.2 mm (**5**).

**Soldier** (Figs 1–5). Head capsule pale brown to orange with yellowish vertex and dark brown nasus; nasus paler in basal part and darker in apical part; antenna uniformly blackish, contrasting with head capsule. Pronotum in dorsal view as darker than head capsule. Abdominal tergites are gold orange to pale brown. Coxae are blackish brown; femora and tibiae pale to dark brown.

Head capsule in dorsal view strongly constricted behind antennal sockets, with anterior part excluding nasus much smaller than posterior part in size; median portion of its posterior margin nearly straight with a minute indentation in the middle; dorsal outline (including nasus) in profile weakly concave, while posterior part of head capsule fairly developed. Nasus in dorsal view more than half as long as receiving head capsule, basal part much wider than tip. Antenna with 14 segments; third segment longer than fourth, while fourth and fifth segment are nearly equal, 6^th^–14^th^ gradually decreasing in length. Pronotum in dorsal view with anterior margin moderately indented in middle and posterior margin roundly convex.

**Figures 6–13.**
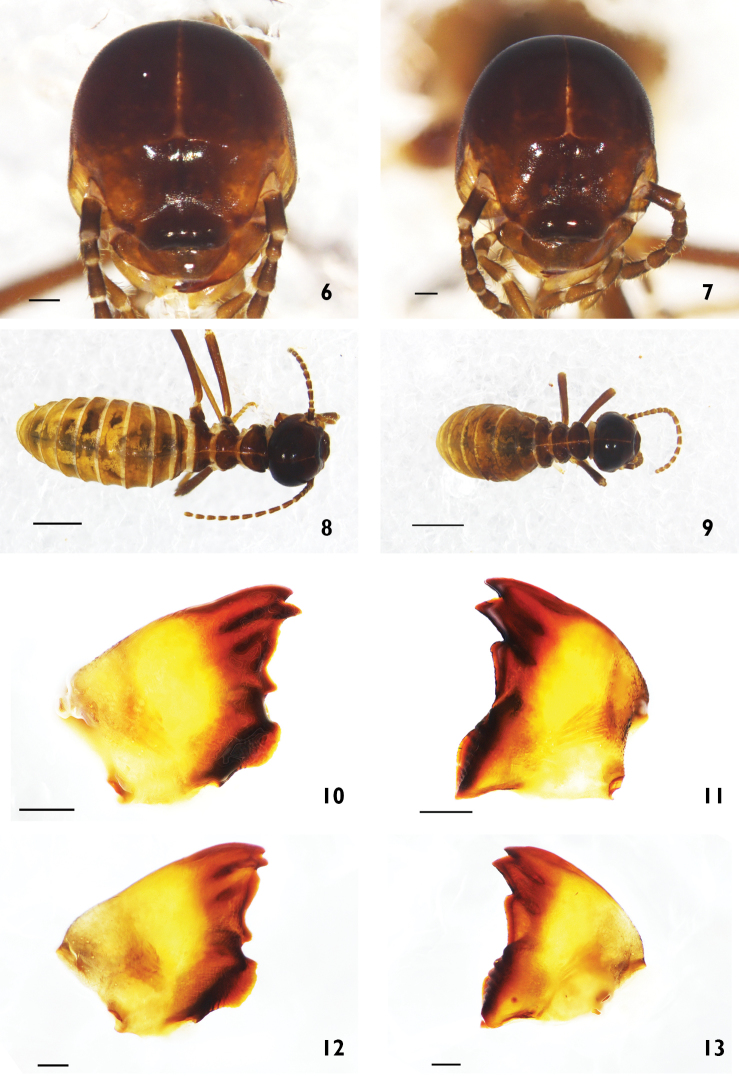
Worker of *Hospitalitermes nigriantennalis* sp. n. Large workers (**6, 8, 10–11**), small worker (**7, 9, 12–13**). Left (**10, 12**) and right (**11, 13**) mandibles. Worker head in dorsal view (**6, 7**), worker habitus in dorsal (**8, 9**). Scale bars: 0.3 mm (**6**), 0.2 mm (**7**), 0.6 mm (**8, 9**), 0.1 mm (**10, 11**), 0.05 (**12, 13**).

**Worker** (Figs 6–13). Dimorphic. *Largest workers*. Head capsule dark brown to black; epicranial suture pale brown; fontanel brown to dark brown; labrum pale brown to dark brown; clypeus blackish brown to black; anteclypeus dark brown; antenna dark brown to blackish. Antenna consisting of 15 segments; third segment longer than fourth, while the fourth segment is slightly shorter than fifth, 6^th^–15^th^ gradually increasing in length. Left mandible: apical tooth clearly shorter than first marginal tooth; anterior edge of first marginal tooth distinctly longer than posterior edge; second marginal tooth absent, incorporated into cutting edge between first and third marginal teeth; third marginal tooth smaller than first marginal tooth, weakly protruding from cutting edge and separated from molar prominence by a distinct gap; fourth marginal tooth retracted, completely hidden behind molar prominence. Right mandible: first marginal tooth with anterior edge down-curved; second marginal tooth weakly recognized and separated from much larger first marginal tooth; posterior edge of second marginal tooth straight; outline of molar plate weakly visible; cockroach notch of molar plate absent.

**Figure 14.**
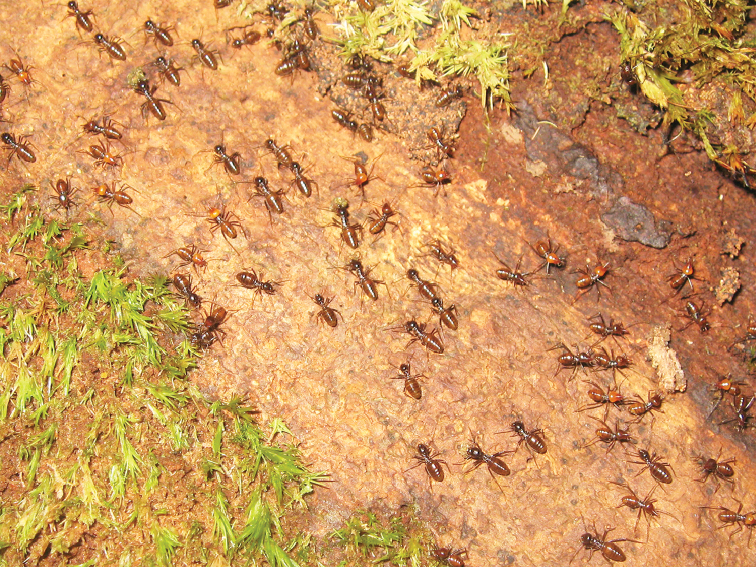
Foraging party of soldiers and workers of *Hospitalitermes nigriantennaris* sp. n. on the forest floor.

**Figure 15.**
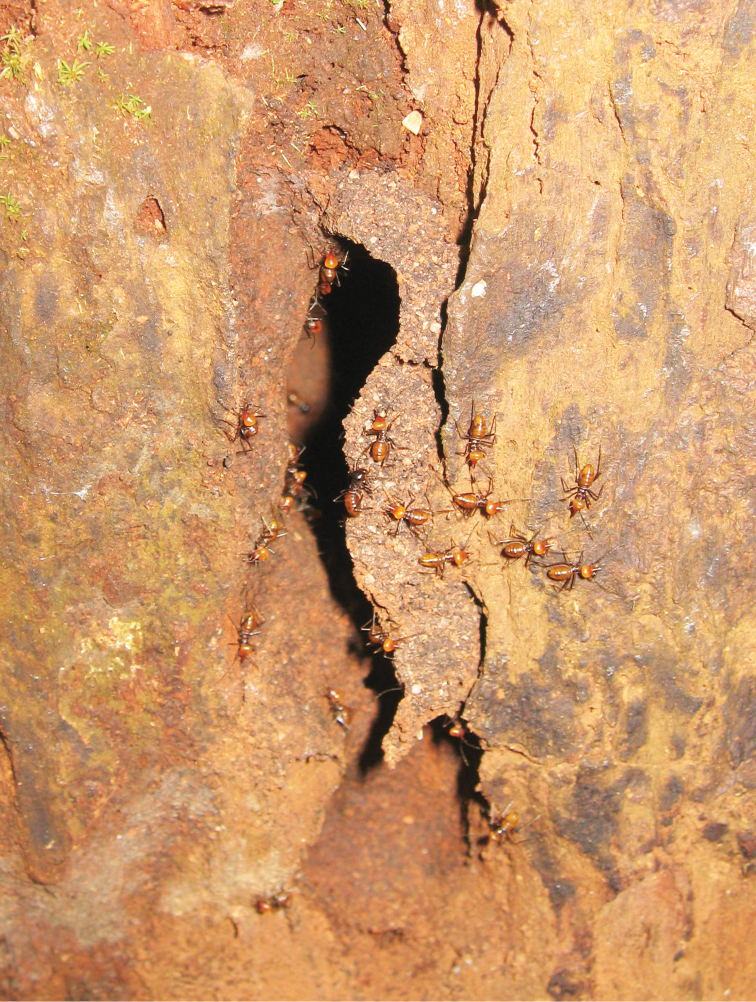
Nest of *Hospitalitermes nigriantennalis* sp. n. inside a standing dead *Shorea* sp. trunk.

##### Comparisons.

*Hospitalitermes nigriantennalis* sp. n. is separated from the other species from Indo-Malayan and Austro-Malayan sub-regions by its peculiar coloration in the soldier. Specifically, *Hospitalitermes nigriantennalis* has prominent black antennae and palps that contrast with the pale head capsule. In other species, the head capsule is uniformly dark and does not contrast with the dark antennae. Further, by morphology *Hospitalitermes nigriantennalis* can be distinguished from other regional congeners *Hospitalitermes rufus*, *Hospitalitermes hospitalis*, *Hospitalitermes medioflavus*, *Hospitalitermes moluccanus* Ahmad, 1947, *Hospitalitermes ferrugineus* (John, 1925), *Hospitalitermes lividiceps*, *Hospitalitermes diurnus* Kemner, 1934, and *Hospitalitermes seikii* Syaukani, 2010 by comparing the head capsule; in dorsal view the posterior margin and the median portion of head capsule of *Hospitalitermes nigriantennalis* are elongated. In other species the head capsule is less elongated, rounded.

Likewise, in lateral view, the dorsal outline (including nasus) in profile weakly separate this new species from congeners (e.g., *Hospitalitermes umbrinus*, *Hospitalitermes birmanicus* (Snyder, 1934), *Hospitalitermes bicolor* (Haviland, 1898), *Hospitalitermes monoceros* (Koenig, 1779), *Hospitalitermes papuanus* Ahmad, 1947, *Hospitalitermes jepsoni* (Snyder, 1934) and *Hospitalitermes krishnai* Syaukani, 2011 by its elongate form.

##### Material examined.

Holotype: soldier collected from a mass processional column on the forest floor (leaving nest to feeding sites) in an undisturbed lowland rain forest (250 m in altitude), Pararawen Nature Reserve (0°38’13”S; 114°41’10”E), North Barito, Central Kalimantan, Borneo. The nest was located in soil at the base of a dead standing tree (*Shorea* sp.), 6 m in height. Syaukani leg. 22.vi.2014. Colony code: SY-2014-Pararawen-0036. Other material from the same locality: SY-2014-Pararawen-C0045, C0051, C0052, C0059 (collected from nests at the base of standing tree), SY-2014-Pararawen-C0019, C0037, C0043 (collected from a processional column *en masse*). The holotype is deposited at Museum Zoologicum Bogoriense (MZB), Cibinong, Indonesia. Paratypes: soldiers and workers from C0019, C0036, C0037, C0043, C0045, C0051, C0052; will be depository at MZB, the Natural History Museum, London (UK), Natural History Museum Vienna (Austria), Syiah Kuala University, Darussalam, Banda Aceh (Indonesia), the Kitakyushu Museum of Natural History and Human History (Japan), and the American Museum of Natural History, New York (USA).

##### Etymology.

This species is named after the blackish antennae in the soldier caste.

##### Biological observation.

With the discovery of this new species, the total number of *Hospitalitermes* species increased to eight for the island of Borneo. This species foraged above the ground and seemed to prefer leaf litter, which may afford some protection from predators. However, when huge logs or fallen trees disrupt this cover, the soldiers can be seen running in a zigzag pattern along the column edges. This observation of soldier behavior is consistent with observations by Hoare and Jones (1998) who reported this response in *Longipeditermes longipes* as a response to disturbance. The strong dimorphism in coloration between the soldier and worker castes is peculiar among the members of this genus, and distinguishes this species. Finally, *Hospitalitermes nigriantennalis* has a dimorphic worker caste.

## Supplementary Material

XML Treatment for Hospitalitermes nigriantennalis
